# The genetic variants in the PTEN/PI3K/AKT pathway predict susceptibility and CE(A)F chemotherapy response to breast cancer and clinical outcomes

**DOI:** 10.18632/oncotarget.15690

**Published:** 2017-02-25

**Authors:** Xiang Li, Ruishan Zhang, Zhuangkai Liu, Shuang Li, Hong Xu

**Affiliations:** ^1^ Department of Breast Cancer, Cancer Hospital of China Medical University, Liaoning Cancer Hospital & Insititute, Dadong District, Shenyang City, Liaoning Province, 110042, P.R.China

**Keywords:** breast cancer, PTEN/PI3K/AKT pathway, genetic polymorphisms, susceptibility, prognosis

## Abstract

The PI3K/PTEN/AKT pathway play a critical role in balancing cell growth and death. Epidemiologic studies suggested that mutations of the PI3K/PTEN/AKT pathway genes are associated with cancer risk, yet no data are available for *PTEN* rs701848, *PIK3CA* rs2699887, and *AKT1* rs2494752 polymorphism and breast cancer(BC) risk. A case-control study was performed in 920 BC patients and 908 healthy controls using the TaqMan assay method. Overall, individuals with *PTEN* rs701848 TC, CC and TC/CC genotypes showed significant increased BC risk (*P*=0.043, *P*=0.002, *P*=0.008, respectively), and the C allele carriers had a 1.224-fold significantly increased risk of developing BC (*P*= 0.003). Moreover, a higher frequency of *AKT* rs2494752 AG genotype was observed among cases (*P*=0.045). Individuals harboring rs2494752 AG/AA genotype had a vital increased susceptibility to BC in the dominant model (*P*=0.039). More importantly, *AKT1* rs2494752 GG genotype showed significantly rates of response to NCT chemotherapy (*P*=0.048). Furthermore, *AKT1* rs2494752 AG genotype carriers showed significantly shorter DFS time, and GG genotype as the independent prognostic factor (DFS: adjusted HR=1.523, 95% CI=1.012-2.293, *P*=0.044; OS: adjusted HR=2.321, 95% CI=1.281-4.204, *P*=0.005). Moreover, MDR analysis consistently revealed that the combination of 3 selected SNPs and 7 known risk factors represented the best model to predicting BC prognosis. The luciferase assay showed that the G allele of rs2494752 significantly increased *AKT1* promoter activity. These results suggest that *PTEN* rs701848 and *AKT1* rs2494752 polymorphisms might be a candidate pharmacogenomic factor to assess the susceptibility of BC and response and prognosis prediction for interindividualized CE(A)F chemotherapy in BC patients.

## INTRODUCTION

Breast cancer (BC) is the leading cause of cancer-related death in women aged 20 to 59 years, with an estimated 231,840 new cases of invasive BC among women in the USA during 2015 [1, 2]. BC alone is expected to account for 29% of all new cancers in women [3]. In China, the incidence of BC is growing at a rate of 3% per year, and the mortality rate increased by 38.9% in cities. Up to now, observational studies have revealed that breast cancer is a complex disease caused by environmental exposures and genetic factors such as germline mutations in a multi-step process of breast carcinogenesis. Recent numerous genomic studies reported that breast cancer consists of a complex biological process with patient-specific genetic variations [4]. The single nucleotide polymorphisms (SNPs) in many candidate genes, especially in signal transduction pathways including the phosphatase and tensin homolog deleted on chromosome10 (pTEN)/phosphatidylinositol-4,5- bisphosphate 3-kinase catalytic subunit alpha (PI3K)/AKT serine/threonine kinase 1(AKT) signaling pathway, often contribute to the origin, propagation, and treatment responses of a cancer and prognosis [5–8]. Therefore, improving the treatment efficacy and prognosis prediction requires a better understanding of the genetic risk factors that involve in the breast cancer carcinogenesis.

The PI3K signaling pathway is one of the most important kinase cascades and exerts its function through its downstream effector AKT in regulating various cell functions including cell proliferation, migration, apoptosis, and angiogenesis. Mutations and dysregulation in this pathway results from mutations or altered expression of an upstream regulator of the AKT activity [9]. Phosphatase and tensin homolog deleted on chromosome 10 (PTEN) dephosphorylates the lipid phosphatidylinositol-3,4,5-triphosphate (PIP3), which is the product of PI3K [10]. The over activation or constitutive activation of PI3K as well as the loss of PTEN function results in the accumulation of cellular PIP3 and its activated downstream effector AKT. More evidences showed that genetic variations of the genes related to this pathway facilitate carcinogenesis and affect individuals’ susceptibility and prognosis to many cancers [11–14]. One study investigated *PTEN* rs701848, *PIK3CA* rs2699887 polymorphisms in a case-control study that consisted of 780 colorectal cancer patients and 764 cancer-free controls, and observed that *PTEN* rs701848 variants were associated with colorectal cancer risk and prognosis of CRC patients treated with FOLFOX regimen [12]. In another study of gastric cancer, Wang *et al*. evaluated five selected SNPs located in the *AKT* promoter region and found that the rs2494752 AG/GG variant genotypes were associated with increased gastric cancer risk, and consequentially the luciferase activity was increased in the patients carrying rs2494752 G allele [13]. Zhu *et al*. investigated seven SNPs in the PI3K/PTEN/AKT/mTOR signaling pathway in 199 advanced non-small cell lung cancer patients, and observed significant associations between platinum-based chemotherapy response and *AKT1* rs2494752 AG genotype [14].

Previous studies have investigated associations between genetic variations in PTEN/PI3K/AKT signaling pathway and cancer risk [15–18], however, the association between *PTEN* rs701848, *PIK3CA* rs2699887, and *AKT1* rs2494752 polymorphisms and sporadic BC in Chinese population has not been investigated yet. In light of the critical role of the PTEN/PI3K/AKT pathway in maintaining proper cellular function, it is possible that some candidate functional SNPs of genes which located in the 5′-untranslated regions (5′UTR) and 3′ UTRs of this pathway may have an effect on susceptibility and response to chemotherapy and prognosis of breast cancer. Therefore, we conducted a hospital-based case-control study of 920 BC patients and 908 cancer-free controls to evaluate associations between SNPs rs701848 located in the 3′UTR of *PTEN*, *PIK3CA* rs2699887, and rs2494752 located in the 5′UTR of *AKT1* and breast cancer risk and response to CE(A)F regimen and clinical outcomes in a Chinese Population.

## RESULTS

### Characteristics of study population

The baseline characteristics and clinical variables of breast cancer patients and cancer-free controls are summarized in Supplementary Table 2. The study included 920 patients with pathologically confirmed breast cancer and a group of 908 age- and gender- matched cancer-free healthy controls. The age was matched between breast cancer patients (range: 24-75 years; median: 52 years) and controls (range: 22-79 years; median: 52 years). There were no significant differences in the distributions of age and menopausal status between cases and controls (*P*=0.676 and *P*=0.682, respectively). Among the cases, 89.4% of CRC patients were invasive ductal cancer (IDC) of breast origin, and in the clinical stage I or II (63.7%). In this cohort, 204 cases underwent preoperative NCT (CE(A)F regimen), and 67.5% of the patients received postoperative anthracycline-based chemotherapy. The clinical variables of age, menopausal status, and first-degree family history of cancer were adjusted for any residual confounding effects in later logistic regression analyses.

### Genotypes in BC cases and controls

Table 1 summarized thefrequencies of allelic and genotype distribution for *PTEN* rs701848, *PIK3CA* rs2699887, and *AKT1* rs2494752 in both 920 cases and 908 controls. The observed genotype frequencies of these three SNPs in the control group all conformed well to Hardy-Weinberg equilibrium (*P*>0.05). As shown in Table 2, there was a significant difference in the genotype distribution of the *PTEN* rs701848 and *AKT1* rs2494752 polymorphisms between the breast cancer patients and the non-cancer controls. For *PTEN* rs701848, the allele and genotype distributions were statistically different between BC cases and controls (TC vs. TT: adjusted OR= 1.254, 95% CI=1.006-1.562, *P*=0.043; CC vs. TT: adjusted OR=1.535, 95% CI=1.167-2.018, *P*= 0.002). Furthermore, a significant increased BC risk was observed in the dominant model (TC/CC vs. TT: adjusted OR=1.326, 95% CI=1.075-1.634, *P*=0.008), meanwhile decreased risk in the recessive model (TC/TT vs. CC: adjusted OR=0.757, 95% CI=0.600-0.954, *P*=0.018). Moreover, the individual carrying C allele had a significant increased risk of developing BC compared with those carrying T allele (C vs. T: adjusted OR=1.224, 95% CI=1.073-1.396, *P*=0.003). For *AKT* rs2494752, a higher frequency of AG genotype were detected among cases in comparison to the controls (53.4% vs. 49.7%, adjusted OR=1.236, 95% CI=1.005-1.519, *P*=0.045). Based on the dominant model, we further observed that individuals harboring AG/AA genotype had a vital increased susceptibility to BC compared with those carrying GG genotype (adjusted OR=1.231, 95% CI=1.010-1.499, *P*=0.039). However, no significant difference in the frequencies of rs2699887 in the *PIK3CA* gene were observed between the BC cases and controls.

**Table 1:** Frequency distribution of *PTEN/PI3K/AKT* pathway genotypes and their associations with the risk for developing BC

Genotypes	No. of cases	%	No. of controls^†^	%	p^‡^	OR	95% CI
*PTEN* rs701848							
TT	215	24.4	273	30.0		1	
TC	468	53.2	474	52.1	0.043	1.254	1.006-1.562
CC	197	22.4	163	17.9	0.002	1.535	1.167-2.018
Dominant model							
TT	215	24.4	273	30.0		1	
TC/CC	665	77.6	637	70.0	0.008	1.326	1.075-1.634
Recessive model							
CC	197	22.4	163	17.9		1	
TC/TT	683	77.6	747	82.1	0.018	0.757	0.600-0.954
Allele frequency							
T allele	898	51.0	1020	56.0		1	
C allele	862	49.0	800	44.0	0.003	1.224	1.073-1.396
*PIK3CA* rs2699887							
GG	671	76.3	689	75.7		1	
GA	197	22.4	201	22.1	0.955	1.006	0.805-1.258
AA	12	1.4	20	2.2	0.185	0.616	0.299-1.270
Dominant model							
GG	671	76.3	689	75.7		1	
GA/AA	209	23.7	221	24.3	0.791	0.971	0.782-1.206
Recessive model							
AA	12	1.4	20	2.2		1	
GA/GG	868	98.6	890	97.8	0.183	1.625	0.790-3.345
Allele frequency							
G allele	1539	87.4	1579	86.8		1	
A allele	221	12.6	241	13.2	0.541	0.941	0.774-1.144
*AKT1* rs2494752							
AA	271	30.8	322	35.4		1	
AG	470	53.4	452	49.7	0.045	1.236	1.005-1.519
GG	139	15.8	136	14.9	0.183	1.214	0.912-1.617
Dominant model							
GG	139	15.8	136	14.9		1	
AG/AA	741	84.2	774	85.1	0.039	1.231	1.010-1.499
Recessive model							
AA	271	30.8	322	35.4		1	
AG/GG	609	69.2	588	64.6	0.618	0.937	0.724-1.211
Allele frequency							
A allele	1012	57.5	1096	60.2		1	
G allele	748	42.5	724	39.8	0.098	1.119	0.979-1.278
Combined genotypes							
1	68	7.7	273	30.0		1	
2	770	87.5	501	55.1	<0.001	6.170	4.624-8.234
3	42	4.8	136	14.9	0.334	1.240	0.802-1.918
2+3	812	92.3	637	70.0	<0.001	5.118	3.848-6.806

Abbreviations: OR, odds ratio; CI, confidence interval.

^†^The observed genotype frequency among individuals in the control group was in agreement with Hardy-Weinberg equilibrium (p^2^+2pq+q^2^=1: *p*=0.092 for *PTEN* rs701848; *p*=0.299 for *AKT1* rs2494752; *p*=0.243 for *PIK3CA* rs2699887).

^‡^*p* were calculated from 2-sided chi-square tests for either genotype distribution or allele frequency.

Combined genotype: 1:rs701848TT and rs2494752AA; 2: rs701848TC and rs2494752GA, rs701848TC and rs2494752AA, rs701848CC and rs2494752GA; 3: rs701848CC and rs2494752GG.

**Table 2:** Association of *PTEN/PI3K/AKT* pathway genetic polymorphisms with therapeutic response to neoadjuvant CE(A)F chemotherapy

Variables	CR and PR		SD and PD		*p*	OR	95%CI
**No**.	**%**	**No**.	**%**
*PTEN* rs701848							
TT	40	70.2	17	29.8		1	
TC	84	75.7	27	24.3	0.953	0.976	0.431-2.207
CC	40	76.9	12	23.1	0.254	0.582	0.230-1.475
Dominant model							
TT	40	70.2	17	29.8		1	
TC/CC	124	76.1	39	23.9	0.162	0.592	0.284-1.234
Recessive model							
CC	40	76.9	12	23.1		1	
TC/TT	124	73.8	44	26.2	0.631	1.208	0.558-2.613
*PIK3CA* rs2699887							
GG	124	75.2	41	24.8		1	
GA	37	72.5	14	27.5	0.658	1.847	0.129-26.358
AA	3	75.0	1	25.0	0.651	1.843	0.123-27.701
Dominant model							
GG	124	75.2	41	24.8		1	
GA/AA	40	72.7	15	27.3	0.926	1.035	0.499-2.146
Recessive model							
AA	3	75.0	1	25.0		1	
GA/GG	161	74.5	55	25.5	0.651	0.542	0.038-7.682
*AKT1* rs2494752							
AA	45	65.2	24	34.8		1	
AG	86	76.1	27	23.9	0.241	0.528	0.182-1.534
GG	33	86.8	5	13.2	0.048	0.325	0.107-0.992
Dominant model							
GG	33	86.8	5	13.2		1	
AG/AA	131	72.0	51	28.0	0.119	2.270	0.811-6.355
Recessive model							
AA	45	65.2	24	34.8		1	
AG/GG	119	78.8	32	21.2	0.077	0.543	0.276-1.068

Abbreviations: CR, complete response; PR, partial response; PD, progressive disease; SD, stable disease.

^†^*p*, OR and 95% CIs were calculated by unconditional logistic regression adjusted for age, menopausal status, family history of BC, tumor size, clinical stages, lymph node metastasis.

Thereafter, we further investigated the effect of the combined genotypes of *PTEN* rs701848 and *AKT1* rs2494752 on the BC risk. The combined effect were group according to the rs701848TT and rs2494752AA considered as references. The combined effect status 2 or 2+3 were significantly associated with an increased risk of BC observed (adjusted OR (95%CI)=6.170(4.624-8.234), *P*<0.001; adjusted OR (95%CI) =5.118(3.848-6.806), *P*<0.001, respectively) (Table 1).

### Association of PTEN/PI3K/AKT pathway polymorphisms and progression of BC

**Figure 1:**
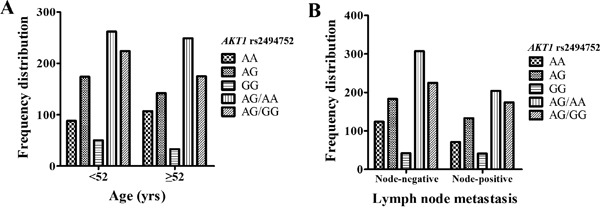
Hstogram and box plots illustrating the frequency distribution of AKT1 rs2494752 polymorphism and stratified clinicopathological features Frequency distribution of *AKT1* rs2494752 genotypes classified by age of onset (<52 years, ≥52 years) **A**. and Lymph node metastasis (Node-negative, Node-positive) **B**.

In the case-only analysis, we further performed stratification analysis to explore the association between polymorphisms of the *PTEN/PI3K/AKT pathway* genes and various clinicopathological characteristics of breast cancer, as shown in Supplementary Table 3 and Figure 1. We found that the frequency (86.8%) of the *AKT1* rs2494752 GA/AA genotypes in BC patients with older ages (≥52 years old) was significantly higher than that those with younger ages (<52 years old) (*P*=0.046, Figure 1A). Furthermore, the frequencies of patients with the *AKT1* rs2494752 GG genotypes in the Lymph node metastasis status (Node-positive, 18.9%) were much higher than in the Lymph node metastasis status (Node-negative, 13.2%) (*P*=0.020, Figure 1B). However, there was no significant association detected between the other two SNPs in *PTEN/PI3K* genes and progression of BC (Supplementary Table 3).

### Effects of PTEN/PI3K/AKT pathway polymorphisms on response to NCT and the survival of BC

Of the 920 BC patients, 220 patients received neoadjuvant CE(A)F chemotherapy. Among the 220 patients, 164 patients were responders and 56 were nonresponders to CE(A)F NCT (Supplementary Table 4). Among the responders, the percentages of *AKT1* rs2494752 AA, AG, GG genotypes were 65.2%,76.1% and 86.8%, respectively (Table 2). GG genotype had significantly rates of response to NCT chemotherapy when compared to the AA genotype (adjusted OR=0.325, 95% CI=0.107-0.992, *P*= 0.048). However, there was no significant association observed between the *PTEN* rs701848 and *PIK3CA* rs2699887 genetic polymorphismsand the response to neoadjuvant CE(A)F chemotherapy (Table 2).

**Figure 2:**
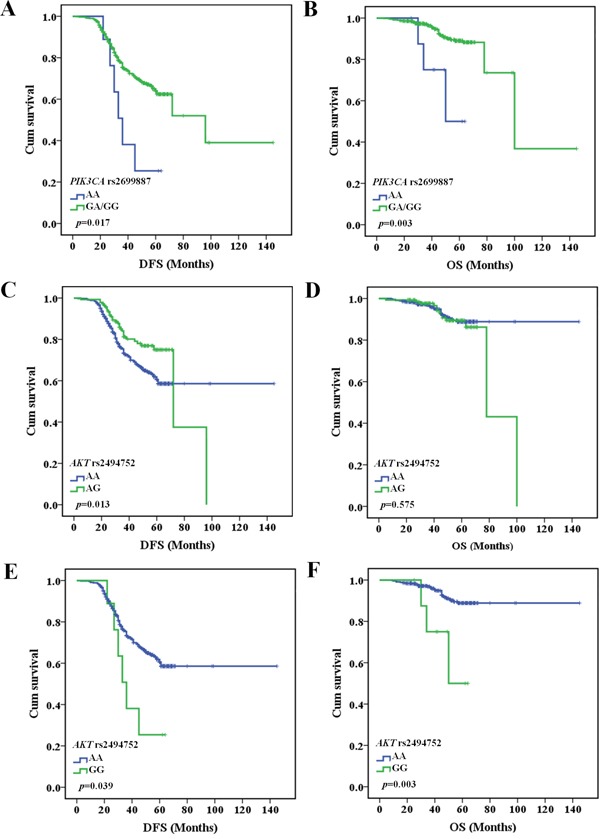
The relationship between the AKT1 rs2494752 polymorphism and BC prognosis according to Kaplan-Meier analysis *PIK3CA* rs2699887 GA/GG genotype had longer DFS **A**. and OS **B**. in BC patients with CE(A)F regimen chemotherapy. *AKT1* rs2494752 AG genotype had shorter DFS **C**., but no correlation with OS **D**. in BC patients after CE(A)F regimen chemotherapy. *AKT1* rs2494752 GG genotype had shorter DFS **E**. and OS **F**. in BC patients after CE(A)F regimen chemotherapy.

To further confirm the predictive effect of the SNPs on prognosis, multivariate Cox regression analysis and Kaplan-Meier analysis were performed to evaluate the correlations between genetic polymorphisms of *PTEN* rs701848, *PIK3CA* rs2699887, and *AKT1* rs2494752 and the prognosis of BC patients after treated with CE(A)F regimen chemotherapy (*n*=594). We observed that rs2699887 genotypes in the *PIK3CA* gene were significantly associated with the DFS and OS of BC patients in the recessive model (log-rank test: *P*=0.017 and *P*=0.003, respectively). In the recessive model, BC patients carrying *PIK3CA* rs2699887 GA/AA genotypes had a significantly longer DFS (median survival time, MST=89 months, 95% CI=73-106 months) and OS time (MST=106 months, 95% CI=80-132 months) in comparison to the carriers who had AA genotype (DFS:MST=40 months, 95% CI=30-51 months, OS: MST=52 months, 95% CI=42-62 months), as shown in Figure 2A, 2B. Multivariate Cox regression analysis further found that *PIK3CA* rs2699887 GA/AA genotypes acted as protective prognostic factors (DFS: adjusted HR=0.384, 95% CI=0.170-0.867, *P*=0.021;OS: adjusted HR=0.192, 95% CI=0.059-0.619, *P*=0.006) adjusted by age, menopausal status, family history of BC, tumor size, clinical stages, lymph node metastasis, outlined in Table 3. Furthermore, Kaplan-Meier analysis revealed that *AKT1* rs2494752 polymorphism was significantly associated with the DFS or OS of BC patients. *AKT1* rs2494752 AG genotype carriers showed significantly shorter DFS time (MST=71 months, 95% CI=58-84 months; Figure 2C) when compared with AA genotype, and verified in multivariate Cox regression model analysis (adjusted HR=0.618, 95% CI=0.417-0.917, *P*=0.017) (Table 3). Moreover, carrying *AKT1* rs2494752 GG genotype had significantly shorter DFS and OS time (DFS: MST=40 months, 95% CI=30-51 months; OS: MST= 53 months, 95% CI=43-62 months; Figure 2D, 2E). Multivariate Cox regression model analysis also evaluated *AKT1* rs2494752 GG genotype as the independent prognostic factor for BC (DFS: adjusted HR=1.523, 95% CI=1.012-2.293, *P*=0.044; OS: adjusted HR=2.321, 95% CI=1.281-4.204, *P*=0.005)(Table 3). However, in this study we did not found significant association between the *PTEN* rs701848 polymorphisms and clinical outcomes in BC patients treated with CE(A)F regimen treatment.

**Table 3:** Multivariate COX regression analysis of *PTEN/PI3K/AKT* pathway polymorphisms and clininopathological features in association with DFS and OS in BC patients with CE(A)F chemotherapy

Variables	DFS			OS		
**HR**	**95% CI**	***p***	**HR**	**95% CI**	***p***
*PTEN* rs701848						
TT	1			1		
TC	1.127	0.785-1.620	0.517	1.048	0.508-2.164	0.899
CC	0.998	0.801-1.244	0.988	1.176	0.791-1.749	0.423
Dominant model						
TT	1			1		
TC/CC	1.092	0.773-1.545	0.617	1.152	0.587-2.263	0.681
Recessive model						
CC	1			1		
TC/TT	1.068	0.748-1.524	0.719	0.702	0.380-1.297	0.259
*PIK3CA* rs2699887						
GG	1			1		
GA	1.033	0.745-1.433	0.846	1.351	0.684-2.668	0.387
AA	1.028	0.813-1.300	0.818	1.340	0.870-2.064	0.185
Dominant model						
GG	1			1		
GA/AA	0.704	0.488-1.016	0.061	1.525	0.829-2.804	0.175
Recessive model						
AA	1			1		
GA/GG	0.384	0.170-0.867	0.021	0.192	0.059-0.619	0.006
*AKT1* rs2494752						
AA	1			1		
AG	0.618	0.417-0.917	0.017	1.305	0.679-2.509	0.425
GG	1.523	1.012-2.293	0.044	2.321	1.281-4.204	0.005
Dominant model						
GG	1			1		
AG/AA	0.948	0.621-1.447	0.804	0.633	0.305-1.315	0.220
Recessive model						
AA	1			1		
AG/GG	1.042	0.761-1.427	0.798	1.463	0.761-2.811	0.254
Age, yrs						
<52	1			1		
≥52	1.379	0.846-2.249	0.198	1.131	0.408-3.137	0.813
Menopausal status						
Premenopausal	1			1		
Postmenopausal	0.653	0.402-1.060	0.085	0.946	0.346-2.588	0.915
Family history of BC						
No	1			1		
Yes	0.510	0.259-1.005	0.052	1.037	0.404-2.660	0.940
Tumor size (cm)						
≤ 2.0	1			1		
>2.0	2.018	1.298-3.137	0.002	2.175	0.892-5.305	0.087
Clinical stages						
I or II	1			1		
III or IV	2.050	1.466-2.867	0.001	3.056	1.571-5.945	0.001
Lymph node metastasis						
Node-negative	1					
Node-positive	0.819	0.595-1.129	0.223	0.937	0.526-1.670	0.888

^†^*p*, HR(95%CI) were assessed using multivariate Cox regression analysis adjusted by age, menopausal status, family history of BC, tumor size, clinical stages, lymph node metastasis.

### Multiple dimension reduction (MDR) analysis

To further evaluate the existence of possible gene, clinical parameters interaction in association with the clinical outcomes, high-order interactions assessed by using the MDR analysis was performed with inclusion of the 3 selected SNPs (i.e., *PTEN* rs701848, *PIK3CA* rs2699887, and *AKT1* rs2494752) and 7 known risk factors (i.e., age at diagnosis, menopausal status, family history, tumor size, clinical stages, lymph node metastasis, histology. In this MDR analysis, 4 risk factors combination were the best model with the highest cross-validation consistency (CVC) and the lowest prediction error in comparison to the one factor model among all 4 risk factors. Moreover, the 10-factor model had a maximum CVC and a minimum prediction error, with the prediction error being statistically significant (Table 4) both in DFS and OS. Taken together, the 10-factor model showed a better prediction prognosis than the 5-factor model and represented the best model to predict BC prognosis for this study.

**Table 4:** MDR analysis for the prediction of prognosis with and without 3 SNPs genotypes

Best interaction models	DFS			OS		
**Cross-validation Consistency**	***P*^†^**	**Training Odds Ratio**	**Cross-validation Consistency**	***P*^†^**	**Training Odds Ratio**
1	100/100	<0.0001	3.25(2.37-4.45)	90/100	<0.0001	8.69(4.97-15.18)
1,2	100/100	<0.0001	3.37(2.46-4.64)	100/100	<0.0001	7.92(4.58-13.69)
1,2,3	100/100	<0.0001	3.48(2.53-4.78)	100/100	<0.0001	8.07(4.67-13.96)
1,2,3,4	100/100	<0.0001	3.40(2.48-4.66)	100/100	<0.0001	8.52(4.93-14.74)
1,2,3,4,5	100/100	<0.0001	3.46(2.53-4.75)	100/100	<0.0001	8.09(4.82-13.59)
1,2,3,4,5,6	100/100	<0.0001	3.53(2.58-4.85)	100/100	<0.0001	7.97(4.61-13.78)
1,2,3,4,5,6,7	100/100	<0.0001	3.32(2.43-4.55)	100/100	<0.0001	7.97(4.75-13.38)
1,2,3,4,5,6,7,8	100/100	<0.0001	3.23(2.34-4.41)	100/100	<0.0001	8.56(5.06-14.46)
1,2,3,4,5,6,7,8,9	100/100	<0.0001	3.55(2.59-4.87)	100/100	<0.0001	8.24(4.76-14.25)
1,2,3,4,5,6,7,8,9,10	100/100	<0.0001	3.83(2.71-5.41)	100/100	<0.0001	9.34(5.48-15.92)

Abbreviations: MDR, multifactor dimensionality reduction.

*P*^†^ value for 1000-fold permutation test.

The best model with maximum cross-validation consistency and minimum prediction error rate was in bold.

labels: 1, age at diagnosis; 2, menopausal status; 3, family history; 4, tumor size; 5, clinical stages; 6, lymph node metastasis;7, histology;8, *PTEN rs701848*; 9, *PIK3CA* rs2699887;10, *AKT1* rs2494752.

### Effect of the rs2494752 polymorphism on AKT1 transcriptional activity

**Figure 3:**
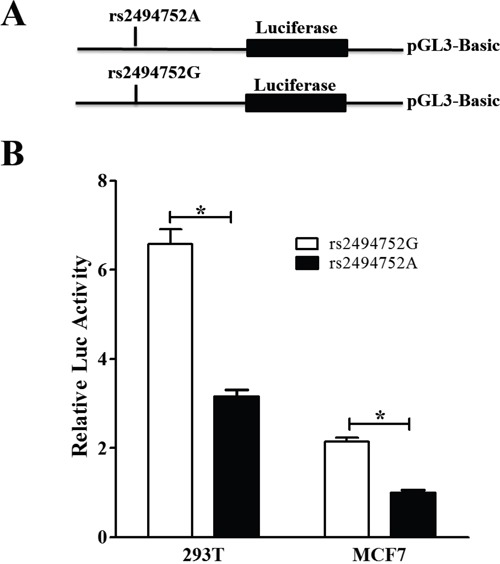
Effect of the rs2494752 polymorphism on the AKT1 promoter activity Schematic representation of reporter plasmids containing the *AKT1* rs2494752 A or G allele **A**., and the two constructs were transiently transfected into the 293T and MCF7 cells **B**. Data were measured as mean ± standard deviation (SD) from 3 separate experiments that were each performed in triplicate (*P<0.05).

We generated reporter gene constructs containing either rs2494752 A or G allele for assess the biological functional effect of rs2494752 SNP on the *AKT1* transcription. The reporter plasmids were transfected into 293T and MCF7 cell lines with. The plasmid containing the rs2494752 G allele showed a significantly higher luc activity expression, in comparison to the A allele, as shown in Figure 3. The results indicated that the SNP rs2494752 G allele in the promoter region had an increased transcriptional activity of the *AKT1* gene.

## DISCUSSION

The PI3K/PTEN/AKT pathway play a critical role in balancing cell growth and death. Mutations or dysregulation of genes involved in this pathway has been associated with invasion, metastasis, and prognosis of a variety of cancers, including BC [19]. Studies confirmed that PIK3CA mutations have been observed as a common occurrence in BC, and the mutations occur at a frequency of 27% to 36% [20]. Additional PI3K pathway alterations in BC include Akt and PTEN mutations, or loss of PTEN protein [21]. Beyond identifying PI3K/PTEN/AKT pathway mutations for understanding breast cancer biology, there are important considerations when this information is used for patient cancer risk prediction and treatment selection. The specific PI3K/PTEN/AKT genetic variations may both impact the treatment response and prognosis. Therefore, in the present study we for the first time performed a case-control study to explore systematically the correlation of PI3K/PTEN/AKT pathway genetic variations with the susceptibility, clinicopathological features, and response to treatment and clinical outcomes of BC patients after CE(A)F regimen.

The present study firstly evaluated the effect of three selected potentially functional SNPs of PI3K/PTEN/AKT pathway on risk of BC. The major finding was a significant association of *PTEN* rs701848 TC/CC genotypes and C allele and *AKT* rs2494752 AG/AA genotype with an increased BC risk under a dominant model in a relatively large cohort (n=920). Our finding that *PTEN* rs701848 polymorphism predicted the individual susceptibility to the development of BC is similar to the results from the studies reported in other tumors such as colorectal cancer, esophageal squamous cell carcinoma (ESCC)[8, 12, 22]. *PTEN* rs701848 SNP is located at 3′-UTR region, which can be targeted by microRNAs, and might alter the strength of microRNAs binding site near the SNP rs701848, thus influencing gene regulation and protein expression. Lin’ study on colorectal cancer (CRC) [12] reported that patients with rs701848 TC/CC genotype in the dominant model and C allele were more likely to be increased CRC risk than carrying CC genotype patients. Xu et al. [8] revealed that the carriers of homozygous mutant of rs701848 polymorphism increased ESCC risk than those with wild-type homozygotes. It is worthy to note that in another 2 case-control studies, one observed that the distribution of genotypes or alleles at *PTEN* rs701848 T/C possessing notably higher proportion in ESCC cases (n=304) than in the controls (n=413) [6], however, the other study did not found any significant differences of the genotype in *PTEN* rs701848 in ESCC patients (n=226) [18]. Although above studies investigated the cancer patients, the different kind of cancer and populations could differ in genetic variations, which can contribute to the discrepancies in these results. Thus, the function of the SNP still needs to be further investigated in future studies. For *AKT* rs2494752 polymorphism, Wang et al [4] found a significantly elevated gastric cancer risk in the patients with the rs2494752 AG/GG variant genotypes. Thereafter, we further investigated the effect of the combined genotypes of *PTEN* rs701848 and *AKT1* rs2494752 on the BC risk, and found 6.170-fold increased risk in rs701848CC and rs2494752GG genotypes. Our results suggested that the *PTEN* rs701848 and *AKT1* rs2494752 polymorphisms may be employed as biomarkers for the prediction of susceptibility of BC. In addition, we analyzed the association between PI3K/PTEN/AKT pathway polymorphisms and clinicopathological features of BC patients. We observed that *AKT1* rs2494752 polymorphism was significantly associated with the age of onset, lymph node metastasis status, suggesting that rs2494752 genotype may be involved in the development and be used as potential biomarker for early screening of the age of onset and the lymph node metastasis status of BC patients.

CE(A)F regimen chemotherapy has been the mainstay of NCT therapy for BC patients, however, the majority of patients will develop resistant, and ultimately recurrence [23, 24]. It is our hope that this study will add to the body of literature for prediction the PI3K/PTEN/AKT pathway genetic polymorphisms lead to individual variation in response to CE(A)F regimen and prognosis. In the present study, we found that the response rate to CE(A)F regimen NCT was higher in the *AKT1* rs2494752 GG genotype carriers when compared to the rs2494752 AA genotype. Thereafter, we further investigated the association of PI3K/PTEN/AKT pathway polymorphisms with the survival time of BC patients treated with CE(A)F regimen. It is worth to note that *PIK3CA* rs2699887 GA/AA genotypes were related to a significantly longer DFS and OS in the recessive model, and multivariate COX and MDR analysis confirmed an independent favorable prognostic value of rs2699887 polymorphism in BC patients. Furthermore, carrying *AKT1* rs2494752 AG or GG genotype showed significantly shorter DFS time when compared with AA genotype, and verified in multivariate Cox and MDR analysis. The rs2494752 SNP is located at the 5′near region of *AKT1* gene, a region predicted to be the potential promoter region of *AKT1* based on sequence alignments, which may disrupt a potential cis-regulatory module affecting gene transcription and translation, and luciferase assay showed that the SNP rs2494752 G allele in the promoter region had an increased transcriptional activity of the *AKT1* gene. Therefore, our results suggested that the polymorphisms in the *AKT1* gene may represent an important potential therapeutic and prognosis for the individual precise treatment in BC patients.

To the best of our knowledge, the present study firstly provide evidence that *PI3K/PTEN/AKT* pathway polymorphism is associated with susceptibility, clinicopathological features and clinical outcome in BC patients treated with CE(A)F regimen in a large and well characterized samples. This data suggest that *PTEN* rs701848 and *AKT1* rs2494752 polymorphisms had a main effect on increasing risk of developing BC, and may be a vital response and prognostic indicator for BC, and employed as candidate biomarker for the prediction of susceptibility and potential chemotherapy in BC patients with CE(A)F regimen in the future.

## MATERIALS AND METHODS

### Study population

This study included 880 patients with breast cancer and 910 age- and gender- matched healthy controls. The patients were recruited at Department of breast cancer, Cancer Hospital of China Medical University, Liaoning Cancer Hospital & Insititute, Liaoning Province, China between July 2009 and December 2015. This cohort was newly diagnosed, histopathologically confirmed and without prior history of other cancers or previous chemotherapy or radiotherapy. Before recruitment, the study was approved by the Research Ethics Committee of China Medical University. The informed consent was obtained from each subject, and detailed personal information on demographic characteristics, smoking and family history of caner were collected by face-to-face interview. Each subject donated 5 mL of venous blood after providing a written informed consent.

The regimens of anthracycline-based chemotherapy contain: CEF (Cyclophosphamide, Epirubicin and 5-Fluorouracil) and CAF (Cyclophosphamide, Adriamycin and 5-Fluorouracil). Patients’ response to anthracycline-based regimen was assessed after the first two or three cycles neoadjuvant chemotherapy (NCT) and determined by the Response Evaluation Criteria in Solid Tumors (RECIST) criteria. All responses were re-evaluated at least 4 weeks after initial assessment. For data analysis, complete responses (CR), partial response (PR) were combined as responders, stable disease (SD) and progressive disease (PD) were grouped as non-responders. Follow-up was performed per two months from the time of enrollment till death or the latest follow-up. Disease–free survival (DFS) was defined as the time between the first day of treatment and an occurrence of recurrence, metastases, death or last follow–up. Overall survival (OS) was calculated as the time between the first day of treatment and death or the last known follow-up.

### DNA extraction and polymorphism genotyping

Genomic DNA was extracted from the peripheral blood (5 mL) by Genomic DNA Extraction Kit Ver.5.0 (Takara Biotechnology (Dalian) Co.LTD, Dalian, China). Three SNPs (MAF≥0.05) located in the 5′ and 3′ UTRs of the *PTEN/PI3K/AKT* pathway genes was selected and genotyped using predesigned TaqMan SNP Genotyping Assays on the ABI 7500 Fast Real-Time PCR platform (Applied Biosystems, Life Technologies Corporation, Foster City, CA, USA). The detailed information of the selected 3 SNPs in PTEN/PI3K/AKT pathway were outlined in Supplementary Table 1. The information about assay conditions, primers, and probes is available upon request. The reaction mixture of 10 mL contained 25 ng genomic DNA, 3.5 mL of TaqMan Genotyping Master Mix, 0.25 mL of the primers and probes mix and 6.25 mL of double distilled water. The amplification was performed under the following conditions: 50°C for 2 min, 95°C for 10 min followed by 45 cycles of 95°C for 15 sec, 60°C for 60 sec. The genotyping rates of these SNPs were all above 95%. For quality control, 5 negative controls were included in each plate and 10% of the samples were randomly selected for repeated genotyping for confirmation; and the results were 100% concordant. All samples were run in duplicates with call rates of >95%, and all output spectra were visually inspected.

### Promoter-reporter plasmids construction and cell lines

To construct the *AKT1* promoter-reporter plasmids, we synthesized the DNA fragment containing either the rs2494752G allele or A allele by the primers of previous publication described by Wang et al [4]. Then, the PCR products were digested with *Kpn*I and *Xho*I and subsequently cloned into the pGL3-basic vector (Promega, Madison, WI, USA) containing the firefly luciferase gene as a reporter. The constructs were all confirmed by DNA sequencing.

The breast cancer cell line (MCF7), Human 293 cell line (293T) were selected to use for the luciferase reporter assay. All these four cell lines were obtained from Chinese academy of sciences (Beijing, China). Cell lines were grown in RPMI-1640 medium supplemented with 10% Fetal Bovine Serum (FBS).

### Luciferase assays

293T and MCF7 cells were cultured in a 24-well culture plates. After 24 h, each well was transfected with 0.8 μg of each *AKT1*-reporter plasmid with the allele A or G using Lipofectamine 2000 (Invitrogen, CA, USA). The pGL3-Basic vector without an insert was used as a negative control. As an internal standard, all plasmids were transfected with 8 ng pRL-SV40 plasmids per well. It contained the Renilla luciferase gene as an internal control for correcting transfection efficiency. After transfections 48 h, cells were lysed with the passive lysis buffer (Promega) and assayed for luciferase expression activity by using the Dual-Luciferase Reporter Assay System (Promega). Three independent transfection experiments were carried out, and each was performed in triplicate.

### Statistical analysis

Data analysis was performed with SPSS software package (Statistical Package for the Social Sciences, version 16.0, SPSS Inc. Chicago, Illinois, USA). All tests were two-sided and statistical significance was set at *P*<0.05. Associations between genetic polymorphisms and breast cancer susceptibility, and clinical variables were analyzed by odds rations (OR) and 95% confidence intervals (CI) using unconditional logistic regression models. The OR and 95%CI were assessed for per-allele, dominant and recessive models. Discrepancies between the expected and observed genotype and allele frequencies were detected by the Hardy-Weinberg equilibrium test (HWE) using a goodness-of-fit chi-square test (*p*^2^+2*pq*+*q*^2^=1). The Kaplan–Meier method and Log-rank test were used to estimate the associations of the DFS and OS with demographic characteristics, and SNPs. Multivariate Cox proportional hazards regression models were applied to obtain the adjusted hazard ratio (HR) and 95% CI for evaluating the effects of genotypes, clinical variables on DFS and OS in breast cancer patients.

## SUPPLEMENTARY MATERIALS AND TABLES



## Abbreviations

*PTEN*: phosphatase and tensin homologue deleted on chromosome 10
*PI3K*: the phosphatidylinositol 3-kinase
*AKT*: Serine/threonine Kinase
SNPs: single-nucleotide polymorphisms
BC: breast cancer
IDC: invasive Ductal carcinoma
ILC: invasive Lobular carcinoma
HWE: Hardy–Weinberg equilibrium
CEF: Cyclophosphamide, Epirubicin and 5-Fluorouracil
CAF: Cyclophosphamide, Adriamycin and 5-Fluorouracil
NCT: neoadjuvant chemotherapy
RECIST: Response Evaluation Criteria in Solid Tumors criteria
CR: complete responses
PR: partial response
SD: stable disease
PD: progressive disease
MST: median survival time
DFS: Disease-free survival
OS: Overall survival
OR: odds ratio
CI: confidence interval
HR: Hazard Ratio
