# Supplementary material for: The genetic variants in the PTEN/PI3K/AKT pathway predict susceptibility and CE(A)F chemotherapy response to breast cancer and clinical outcomes
Source: Oncotarget. 2017 Feb 25;8(12):20252–65. doi: 10.18632/oncotarget.15690 (PMC5386760; doi:10.18632/oncotarget.15690)
Supplement: Supplementary file 1 [file oncotarget-08-20252-s001.pdf]

The genetic variants in the *PTEN/PI3K/AKT* pathway predict susceptibility and CE(A)F chemotherapy response to breast cancer and clinical outcomes

SUPPLEMENTARY MATERIALS

SUPPLEMENTARY TABLES

Supplementary Table 1: Detailed information of the selected SNPs in PTEN/PI3K/AKT pathway

Genes	Full name	SNP	Base change	Chr	SNP location	MAF
PTEN	the phosphatase and tensin homolog deleted on chromosome10	rs701848	T/C	10q23.31	3'-UTR	0.3734
PI3KCA	phosphatidylinositol-4,5-bisphosphate 3-kinase catalytic subunit alpha	rs2699887	G/A	3q26.32	Intron	0.1176
AKT1	AKT serine/threonine kinase 1	rs2494752	A/G	14q32.33	5'-UTR	0.3299

Supplementary Table 2: Baseline characteristics in patients with breast cancer (cases, *n*=920) and cancer-free controls (*n*=908)

Variable	No. of cases	%	No. of controls[†]	%	p[‡]
Total no.	880	100	910	100	
Median age [range], yrs	52[24–75]		51[22–79]		
Age, yrs					
<52	470	53.4	495	54.4	0.676
≥52	410	46.6	415	45.6	
Menopausal status					
Premenopausal	476	54.1	501	55.1	0.682
Postmenopausal	404	45.9	409	44.9	
Family history of BC					
No	797	90.6			
Yes	83	9.4			
Histology					
IDC	787	89.4			
ILC	25	2.8			
Others [‡]	68	7.7			
Tumor size (cm)					
≤ 2.0	197	22.4			
>2	683	77.6			
Clinical stages					
I or II	561	63.7			
III or IV	319	36.3			
Lymph node metastasis					
Node-negative	478	54.3			
Node-positive	402	45.7			
ER status					
Negative	323	36.7			
Positive	557	63.3			
PR status					
Negative	384	43.6			
Positive	496	56.4			
NCT					
CE(A)F regimen	204	23.2			
Postoperative therapeutic regimens					
CE(A)F regimen	594	67.5			
A(E)T or TAC regimen	150	17.0			
T or TP regimen	93	10.6			
Others	43	4.9			

Abbreviations: IDC, invasive ductal carcinoma; ILC, invasive lobular carcinoma; ER, Estrogen receptor; PR, Progesterone receptor; NCT, neoadjuvant chemotherapy; CE(A)F, Cyclophospha- mide, Epirubicin (Adriamycin) and Fluorouracil; NE, Navelbine and Epirubicin; A(E)T, Adriamycin (Epirubicin) and Taxotere; TAC, Taxotere, Adriamycin and Cyclophosphamide; T, Taxotere; TP, Taxotere and Cisplatin.

[†]Determined using a 2-sided chi-square test.

Supplementary Table 3: Association between *PTEN/PI3K/AKT* pathway polymorphisms and clinicopathological features in BC patients

Variables	*PTEN* rs701848					*PIK3CA* rs2699887					*AKT1* rs2494752				
	TT	%	TC/CC	%	$p^{\dagger}$	GG	%	GA/AA	%	$p^{\dagger}$	GG	%	AG/AA	%	$p^{\dagger}$
Age, yrs															
<52	111	23.6	359	76.4	0.547	350	74.5	120	25.5	0.184	85	18.1	385	81.9	0.046
≥52	104	25.4	306	74.6		321	78.3	89	21.7		54	13.2	356	86.8	
Menopausal status															
Premenopausal	116	24.4	360	75.6	0.963	355	74.6	121	25.4	0.206	77	16.2	399	83.8	0.737
Postmenopausal	99	24.5	305	75.5		316	78.2	88	21.8		62	15.3	342	84.7	
Family history of BC															
No	197	24.7	600	75.3	0.541	607	76.2	190	23.8	0.847	124	15.6	673	84.4	0.550
Yes	18	21.7	65	78.3		64	77.1	19	22.9		15	18.1	68	81.9	
Histology															
IDC	193	24.5	594	75.5	0.982	598	76.0	189	24.0	0.816	127	16.1	660	83.9	0.148
ILC	6	24.0	19	76.0		19	76.0	6	24.0		6	24.0	19	76.0	
Others	16	23.5	52	76.5		54	79.4	14	20.6		6	8.8	62	91.2	
Tumor size (cm)															
≤ 2.0	53	26.9	144	73.1	0.359	147	74.6	50	25.4	0.542	34	17.3	163	82.7	0.523
>2	162	23.7	521	76.3		524	76.7	159	23.3		105	15.4	578	84.6	
Clinical stages															
I or II	135	24.1	426	75.9	0.736	429	76.5	132	23.5	0.838	92	16.4	469	83.6	0.515
III or IV	80	25.1	239	74.9		242	75.9	77	24.1		47	14.7	272	85.3	
Lymph node metastasis															
Node-negative	105	22.0	373	78.0	0.063	357	74.7	121	25.3	0.235	63	13.2	415	86.8	0.020
Node-positive	110	27.4	292	72.6		314	78.1	88	21.9		76	18.9	326	81.1	
ER status															
Negative	78	24.1	245	75.9	0.882	251	77.7	72	22.3	0.439	52	16.1	271	83.9	0.851
Positive	137	24.6	420	75.4		420	75.4	137	24.6		87	15.6	470	84.4	
PR status															
Negative	91	23.7	293	76.3	0.656	294	76.6	90	23.4	0.848	64	16.7	320	83.3	0.533
Positive	124	25.0	372	75.0		377	76.0	119	24.0		75	15.1	421	84.9	

† *p* value was obtained from two-sided chi-square test.

Supplementary Table 4: Clinicopathological characteristics in patients with NCT

Characteristics	CE(A)F regimen	
	No.	%
Total no.		
Age, yrs		
<52	97	44.1
≥52	123	55.9
Menopausal status		
Premenopausal	121	55.0
Postmenopausal	99	45.0
Family history of BC		
No	214	97.3
Yes	6	2.7
Tumor size (cm)		
≤ 2.0	6	2.7
>2	214	97.3
Histology		
IDC	199	90.5
ILC	6	2.7
Others †	15	6.8
Clinical stages		
I or II	108	49.1
III or IV	112	50.9
Lymph node metastasis		
Node-negative	126	57.3
Node-positive	94	42.7
ER status		
Negative	107	48.6
Positive	113	51.4
PR status		
Negative	137	62.3
Positive	83	37.7
Response to NCT		
CR+PR	164	74.5
SD+PD	56	25.5

Abbreviations: IDC, invasive ductal carcinoma; ILC, invasive lobular carcinoma; ER, Estrogen receptor; PR, Progesterone receptor.
Determined using a 2-sided chi-square test.
†Others contains: invasive cribriform carcinoma, medullary carcinoma, and invasive papillary carcinoma.
